# Supplementary material for: Broad Vitamin B6-Related Metabolic Disturbances in a Zebrafish Model of Hypophosphatasia (TNSALP-Deficiency)
Source: Int J Mol Sci. 2025 Apr 1;26(7):3270. doi: 10.3390/ijms26073270 (PMC11990062; doi:10.3390/ijms26073270)
Supplement: Supplementary file 1 [file ijms-26-03270-s001.zip › Suplemental Figures_alpl_manuscript_IJMS_R1.pdf]

Supplemental Figures

Figure S1

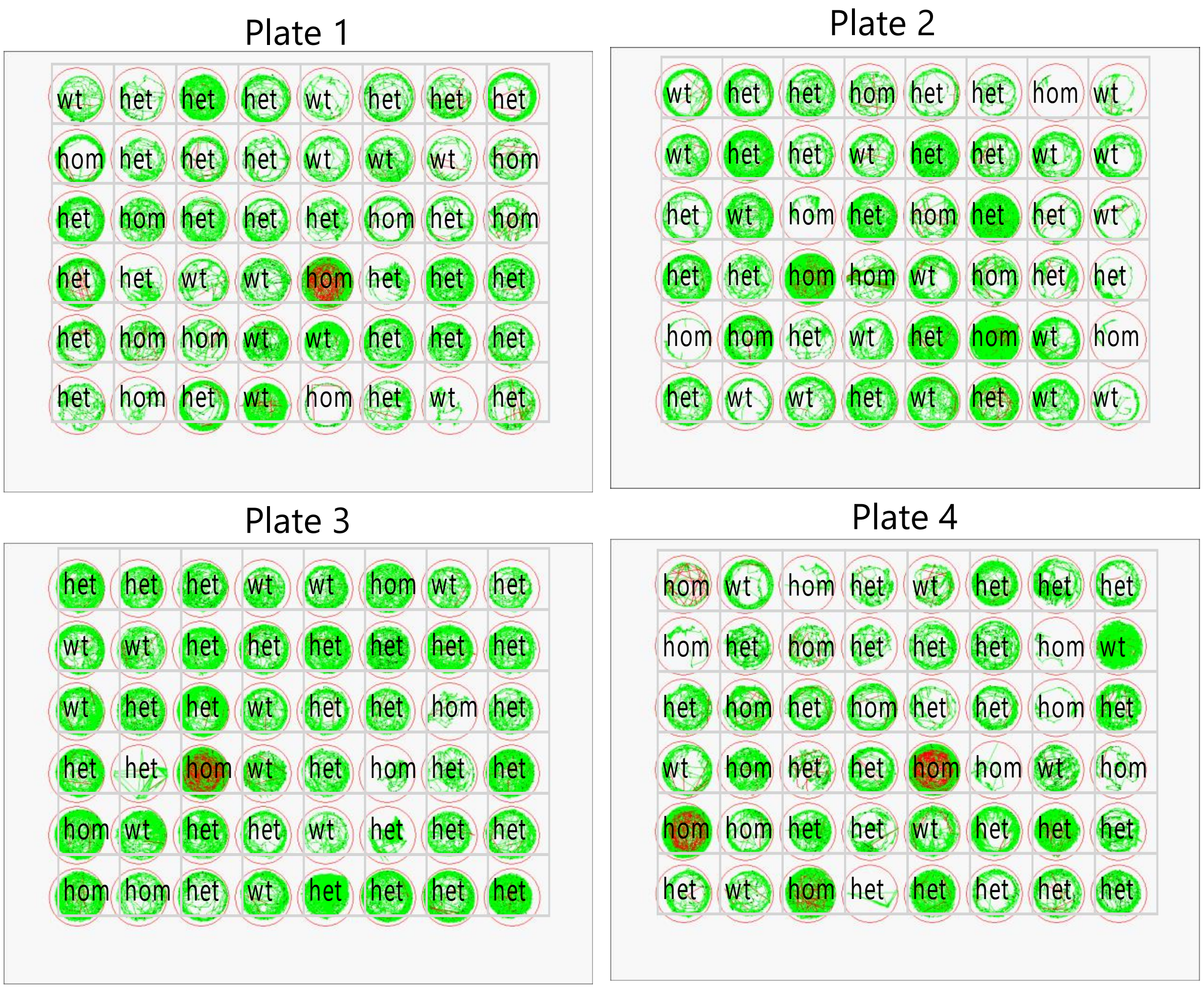

Figure S1. Overview of swimming trajectories of 5 dpf old WT, *alpl*$^{+/-}$, *alpl*$^{-/-}$ zebrafish embryos. Zebrafish embryos were placed in random order (1 embryo/well) in 48-well plates containing 0.5 ml/well embryo medium E3. After 15 min acclimatization, swimming trajectories were recorder for 1 hour at 28 °C in the dark using Zebrabox. In each well the genotype of the embryo is indicated: wt – wild type, het – *alpl*$^{+/-}$, hom - *alpl*$^{-/-}$. Total n=192, WT n=45, *alpl*$^{+/-}$ n=105, *alpl*$^{-/-}$ n=42. Color coding: green – movement speed <30 mm/s (small activity), red – movement speed >30 mm/s (burst), black – no movement (freeze).

Figure S2

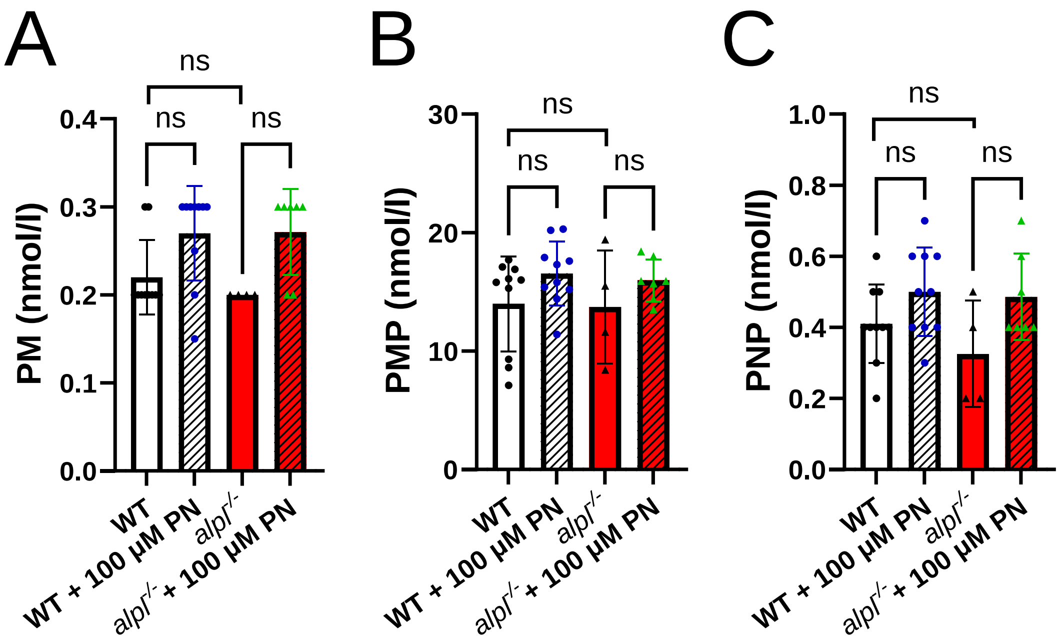

Figure S2. The effects of 72 hours continuous treatment with 100 µM pyridoxine on B_6 vitamers in 5dpf old WT and *alpl*$^{-/-}$ embryos. A) pyridoxamine (PM) concentration, B) pyridoxamine 5'-phosphate (PMP) concentration and C) pyridoxine 5'-phosphate (PNP) concentration. Data are means from n=4-10 pools of 3 embryos ±SD. ****p<0.0001, ns - not significant (p>0.05).

Figure S3

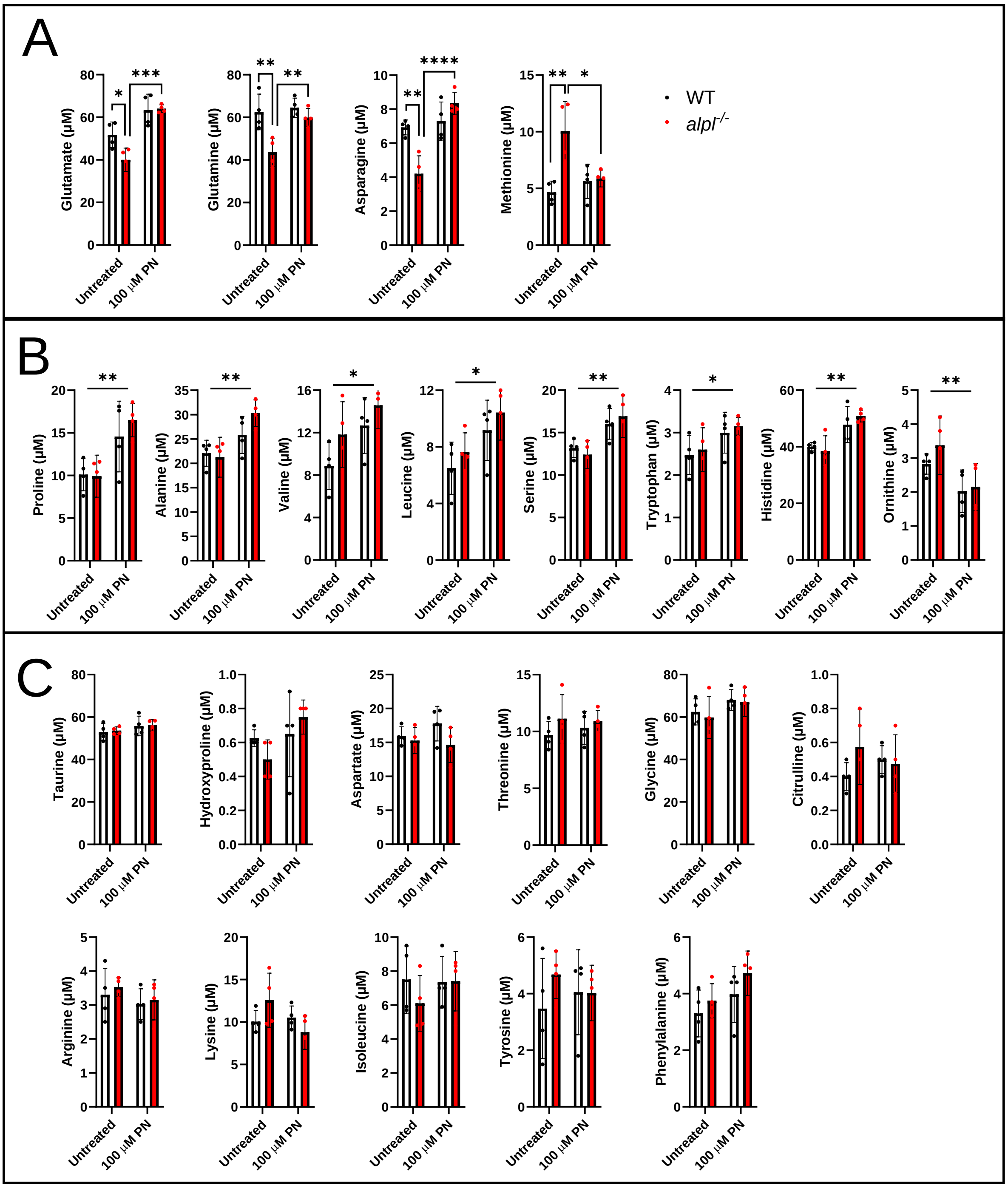

Figure S3. The effects of 72 hours continuous treatment with 100 µM pyridoxine (PN) on amino acid concentrations in 5dpf old WT and *alpl*$^{-/-}$ embryos. A) Amino acids that were significantly changed in *alpl*$^{-/-}$ embryos compared to WT, **B)** Amino acids that were significantly changed in response to PN-treatment (independent of genotype), and **C)** Amino acids that were not significantly affected by genotype or PN-treatment. Data are means from n=4 pools (3 embryos/pull) per genotype and treatment ±SD. ****p<0.0001, ***p<0.001, **p<0.01 and *p<0.05 (comparisons as indicated in the graphs). First, two-way ANOVA was performed for all amino acids to determine the effect of genotype and PN-treatment. In panel B, significant effect of PN-treatment (independent of genotype) is shown. For amino acids shown in panel A, significant

interaction was found between the effect of genotype and PN-treatment. Therefore, one-way ANOVA with Tuckey's multiple comparisons test was performed to compare individual groups, and the significant effects of this test are shown in the graphs.

Figure S4

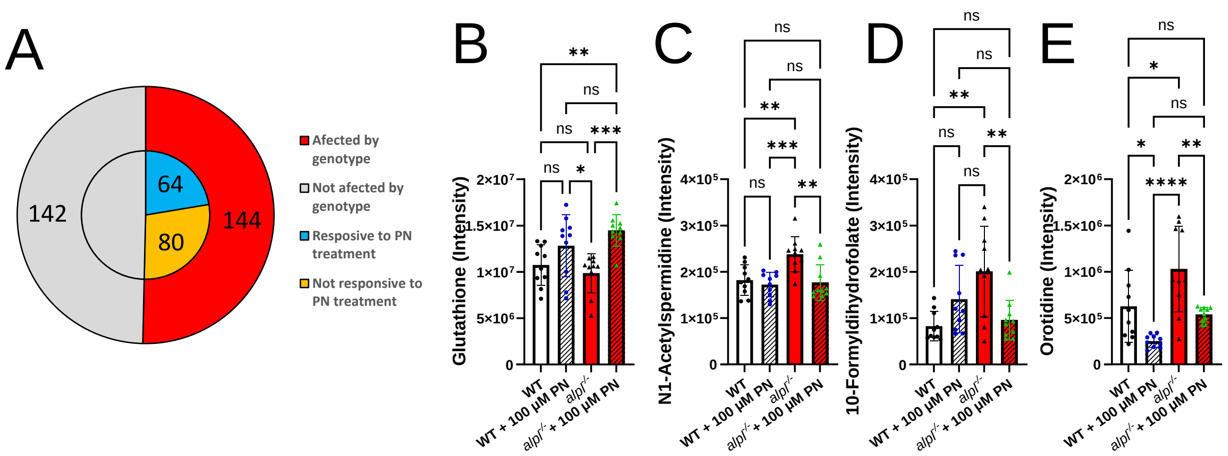

Figure S4. Analysis of DI-HRMS data.

A) Pie chart visualization of one-way ANOVA results showing fractional contribution of metabolites significantly (WT vs. alpl KO p<0.05) and not significantly (WT vs. alpl KO p>0.05) affected by genotype, and responsive to pyridoxine (PN)-treatment (alpl KO+PN vs. alpl KO p<0.05), and not responsive to PN-treatment (alpl KO+PN vs. alpl KO p>0.05).

B) The effects of alpl deficiency and PN-treatment treatment on glutathione levels in 5dpf embryos. Data are means from n=10 per genotype and treatment group ±SD. ***p<0.001, **p<0.01, *p<0.05 and ns – p>0.05; comparisons as indicated in the graphs.

C) The effects of alpl deficiency and PN-treatment on N1-acetylspermidine levels in 5dpf embryos. Data are means from n=10 per genotype and treatment group ±SD. ***p<0.001, **p<0.01 and ns – p>0.05; comparisons as indicated in the graphs.

D) The effects of alpl deficiency and PN-treatment on 10-formylhydrofolate levels in 5dpf embryos. Data are means from n=10 per genotype and treatment group ±SD. **p<0.01 and ns – p>0.05; comparisons as indicated in the graphs.

E) The effects of alpl deficiency and PN-treatment on orotidine levels in 5dpf embryos. Data are means from n=10 per genotype and treatment group ±SD. ****p<0.0001, **p<0.01, *p<0.05 and ns – p>0.05; comparisons as indicated in the graphs.

Figure S5

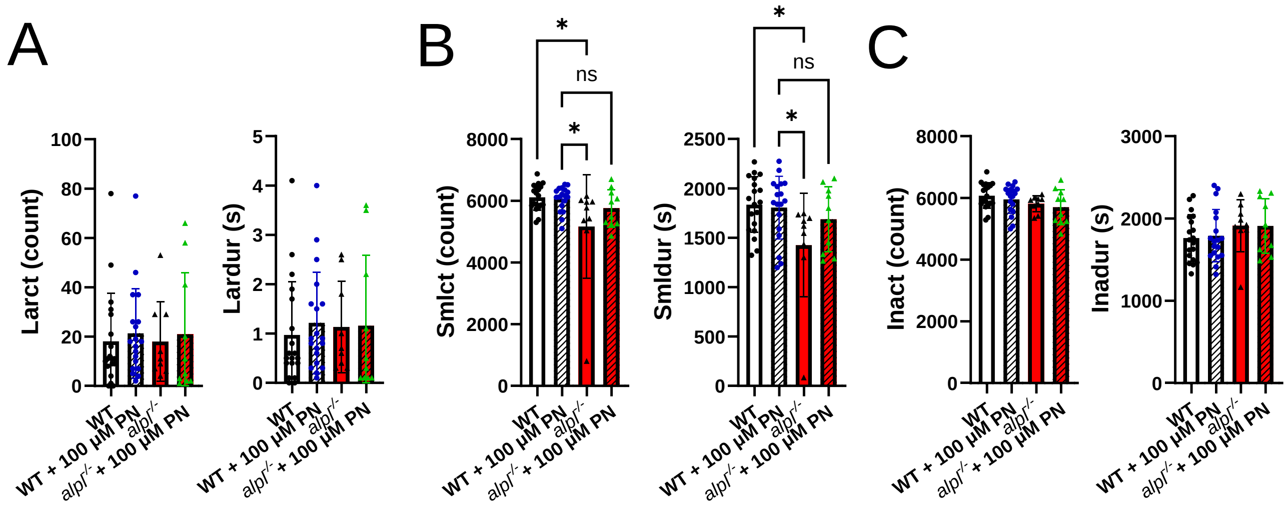

Figure S5. Locomotion parameters in untreated and 100 µM pyridoxine (PN)-treated 10 dpf zebrafish larvae. A) Burst activity (movement speed >30 mm/s) count and duration. **B)** Small activity (movement speed <30 mm/s) count and duration. **C)** Inactivity (no movement) count and duration. The locomotion was recorded using Zebrabox in the tracking mode for 1 hour at 28°C in the dark. Data are means from n=9-19 larvae per genotype and treatment condition ±SD. *p<0.05; comparisons as indicated in the graphs.

Figure S6

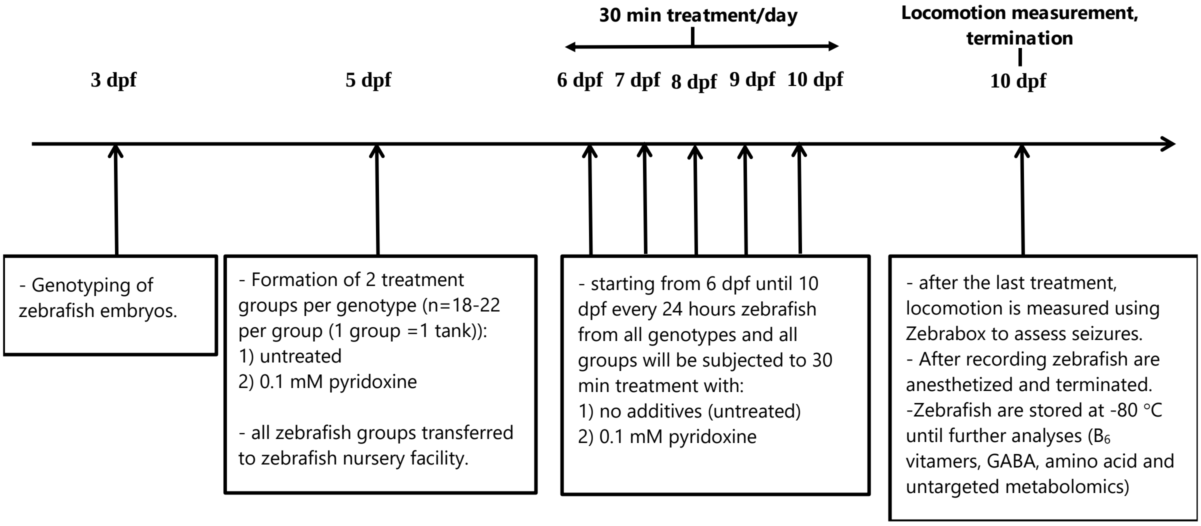

Figure S6. Overview of the experimental setup with 10 dpf old zebrafish larvae.
