# Supplementary material for: Broad Vitamin B6-Related Metabolic Disturbances in a Zebrafish Model of Hypophosphatasia (TNSALP-Deficiency)
Source: Int J Mol Sci. 2025 Apr 1;26(7):3270. doi: 10.3390/ijms26073270 (PMC11990062; doi:10.3390/ijms26073270)
Supplement: Supplementary file 1 [file ijms-26-03270-s001.zip › Table S1.pdf]

Table S1. Oligonucleotide sequences.

Oligo name	Application	Oligo sequence
alpl_ex5_chr11:28870990_S1	sgRNA	GGTGACCTCGTTCCCCTGAG
alpl_ex6_chr11:28871789_S2	sgRNA	GGCATAAGCAGCACTCGGGG
alpl_ex5_FW	Genotyping PCR	ACTGCCACAGCTTTCCTC
alpl_ex5_RV	Genotyping PCR	GCAGAAAGGCATTATAGCAG
alpl_4/5_qPCR_FW	qPCP	GGGCTAAAGACGCAGGCAAA
alpl_4/5_qPCR_RV	qPCR	CCACACAGTGGGCATAAGCA
b-actin_qPCR_FW	qPCR	CGAGCTGTCTTCCCATCCA
b-actin_qPCR_RV	qPCR	TCACCAACGTAGCTGTCTTTCTG
